# Function of Excitatory Periaqueductal Gray Synapses in the Ventral Tegmental Area following Inflammatory Injury

**DOI:** 10.1523/ENEURO.0324-22.2022

**Published:** 2022-12-19

**Authors:** Claire Elena Manning, Michael Fritz, Julie Ann Kauer

**Affiliations:** Nancy Pritzker Laboratory, Department of Psychiatry and Behavioral Sciences, Stanford University, Stanford, California 94305-5101

**Keywords:** carrageenan, glutamate, pain, periaqueductal gray, ventral tegmental area

## Abstract

Manipulating the activity of ventral tegmental area (VTA) dopamine (DA) neurons can drive nocifensive reflexes, and their firing rates are reduced following noxious stimuli. However, the pain-relevant inputs to the VTA remain incompletely understood. In this study, we used male and female mice in combination with identified dopamine and GABA neurons in the VTA that receive excitatory inputs from the periaqueductal gray (PAG), a nexus of ascending pain information. We tested whether PAG–VTA synapses undergo functional plasticity in response to a pain model using optical stimulation in conjunction with slice electrophysiology. We found that acute carrageenan inflammation does not significantly affect the strength of excitatory PAG synapses onto VTA DA neurons. However, at the PAG synapses on VTA GABA neurons, the subunit composition of NMDA receptors is altered; the complement of NR2D subunits at synaptic sites appears to be lost. Thus, our data support a model in which injury initially alters synapses on VTA GABA neurons. Over time, these alterations may increase tonic inhibition of VTA DA neurons leading to their reduced firing.

## Significance Statement

Following a focal injury, the firing rate of dopamine neurons of the ventral tegmental area (VTA) decreases, despite a lack of direct innervation from the periphery. Here we assess the functional changes between a primary node of nociceptive output, the periaqueductal gray (PAG), and the VTA after peripheral inflammation. We find that synaptic strength at PAG-to-VTA dopamine neuron synapses is unaffected following inflammatory injury, but find a change in subunit composition of NMDARs at PAG synapses on the inhibitory neurons of the VTA.

## Introduction

Pain and analgesia are complex phenomena regulated at multiple sites in ascending and descending nociceptive pathways. Much has been uncovered at the level of the periphery and spinal cord itself, but the role of supraspinal structures in shaping pain perception and analgesia is not fully understood.

The ventral tegmental area (VTA) contains predominantly dopaminergic neurons and a prominent minority of GABAergic neurons, as well as a small number of glutamatergic neurons (Nair-Roberts et al., 2008). During noxious pain events, most dopamine (DA) neurons are inhibited (Mirenowicz and Schultz, 1996; Ungless et al., 2004; Yang et al., 2021), while GABA neurons are excited (however, for discussion of noxious phasic excitation in DA neurons, see Brischoux et al., 2009). The intrinsic excitability of most DA neurons is unchanged in chronic pain models (Huang et al., 2019), despite reduced firing rates *in vivo* (Ren et al., 2016; Watanabe et al., 2018) and a significant increase in inhibitory synaptic input to DA neurons (Markovic et al., 2021; Yang et al., 2021). Furthermore, silencing of VTA DA neurons reduces the strength or time needed for a stimulus to elicit nociceptive reflexes, while stimulation of these neurons during neuropathic pain models drives analgesic behavior (i.e., increased stimulation is required to elicit a nociceptive reflex and an increase is seen in time spent in a chamber associated with pain; Navratilova et al., 2012; Watanabe et al., 2018; de Jong et al., 2019; Taylor et al., 2019b; Waung et al., 2019; Sun et al., 2021). Within the VTA, opiate drugs act mainly through μ-opioid receptors located on GABA neurons of the VTA where they act to disinhibit DA neurons (Johnson and North, 1992). Together, this information suggests a simple model in which inhibitory inputs onto dopamine neurons are the primary drivers of the neuroadaptations following pain experience. However, very few studies have considered functional changes in excitatory inputs onto either dopamine or GABA VTA neurons during a pain model. Furthermore, the source of nociceptive circuit inputs to VTA neurons are still being identified.

One input that is potentially relevant to nociception is the periaqueductal gray (PAG). A subset of spinal cord neurons carrying nociceptive information synapse directly within the PAG (Basbaum et al., 2009), making it a primary site for regulation of ascending nociceptive information. Furthermore, the PAG is a known nexus of ascending nociceptive information and opiate-mediated analgesia (Lewis and Gebhart, 1977; Mendes-Gomes et al., 2011; Campion et al., 2016). The PAG contains nonoverlapping populations of glutamatergic and GABAergic neurons, as well as a minority of monoaminergic neurons (Suckow et al., 2013; Samineni et al., 2017). All of these cell types have been implicated in pain modulation and the affective experience of pain, and these roles appear to depend on their projection targets and actions. For example, driving inhibitory PAG neurons facilitates nociception (Samineni et al., 2017), and inhibitory PAG projections to the amygdala contribute to fear learning and anxiety behaviors (Lowery-Gionta et al., 2018). Monoaminergic PAG neurons are critical in pain processing (Taylor et al., 2019a) in part through their projections to the amygdala (Li and Kash, 2019), whereas locally acting monoaminergic PAG neurons can drive freezing behavior (Vaaga et al., 2020). Driving excitatory PAG neurons suppresses nociception, as well as anxiety-related behavior (Samineni et al., 2017; Taylor et al., 2019a). Together, these findings emphasize a pivotal role for the PAG and its afferents in pain experience.

Relatively little is known about the behavioral consequences of driving PAG projections to the ventral tegmental area. PAG afferents make both excitatory and inhibitory (but not monoaminergic projection) synapses on identified GABA and DA neurons of the VTA (Omelchenko and Sesack, 2010; Suckow et al., 2013; Ntamati et al., 2018; Waung et al., 2019). Furthermore, the inhibitory projections from the PAG to VTA DA neurons can undergo a unique opiate-sensitive long-term potentiation (LTP; St Laurent et al., 2020). This further indicates that PAG–VTA projections could be an important nexus of supraspinal functional changes during pain and pain relief. However, there is a dearth of evidence investigating this intriguing hypothesis. To date, and to our knowledge, only two behavioral studies have tested manipulation of the PAG–VTA circuit. The first reported that driving all PAG-to-VTA afferents is aversive, supporting conditioned place aversion. However, inhibition of these pathways is only reinforcing when animals are in a pain state (Waung et al., 2019). This finding further supports the hypothesis that this pathway undergoes functional plasticity in the context of pain. A second study found that specifically driving inhibitory PAG afferents to the VTA is not reinforcing in naive mice (St Laurent et al., 2020).

Together, these findings indicate a possible role for excitatory PAG–VTA inputs contributing to the negative affect associated with pain. At the synapse level, this could manifest either as a reduction in glutamatergic synaptic strength on VTA dopamine neurons, and/or an increase in glutamatergic synaptic strength on VTA GABA neurons. However, there are no studies on the functional changes in the excitatory PAG–VTA circuit following injury. Here we used an inflammatory pain model to assess changes in functional excitatory neurotransmission in the PAG-to-VTA circuit, specifically analyzing glutamatergic synapses onto both DA and GABA neurons. During inflammatory pain, we found no changes in excitatory synapse strength at PAG–VTA DA neurons, but report subtle changes at synapses on PAG–VTA GABA neurons. After inflammatory injury, the subunit composition of NMDA receptors (NMDARs) in these synapses on VTA GABAergic neurons is altered, with a reduced complement of NR2D subunits.

## Materials and Methods

### Animals

All experiments were performed with approval of protocols from Stanford University Institutional Animal Care and Use Committee. B6J.129S6(FVB)-Slc32a1^tm2(cre)Lowl/^MwarJ (VGat-Cre; catalog #028862; https://www.jax.org/strain/028862), B6.Cg-*Gt(ROSA)26Sor^tm14(CAG-tdTomato)Hze^*/J (Ai14; catalog #007914; https://www.jax.org/strain/007914), and B6.129P2-Pitx3^tm1Mli^/Mmjax (Pitx3-GFP) (catalog #41479; https://www.jax.org/strain/028554) mice were obtained from The Jackson Laboratory and bred at Stanford University. Males and females were both used in experiments. Although we were not sufficiently powered to determine sex differences, we identify the number of samples from males and females in each experiment (Table 1). Mice were housed in a 12 h light/dark (LD) cycle and fed *ad libitum*.

**Table 1:** Classification of sexes used in each analysis

Experiment	Figure	Panels	Control		Experimental		Sum
			Female	Male	Female	Male	
Gq DREADD	1	C–E	3	3	3	3	12
Gi DREADD	1	F–H	3	4	4	2	13
Pain behavior 4 h	2	A–D	6	2	6	1	15
Pain behavior 24 h	2	E, F	2	5	2	6	15
DA neuron PPR	3	C, D	5	14	5	12	36
GABA neuron PPR	3	F, G	9	2	5	6	22
DA AMPA/NMDA	4	B	7	7	1	9	24
DA –70	4	C	9	13	3	15	40
DA +40	4	D	8	7	1	11	27
DA NASPM	4	E	4	8	4	6	22
GABA AMPA/NMDA	5	B	10	2	6	8	26
GABA –70	5	C	13	4	9	11	37
GB +40	5	D	11	3	7	8	29
GABA NASPM	5	E	8	0	2	3	13
GABA GLUN2D UBP	6	B, C	5	5	4	7	21
GABA GLUN2D ifenprodil	6	E, F	5	4	4	7	20

### Intracranial injection surgery

Animals were deeply anesthetized with a mixture of ketamine (9 mg/kg) and xylazine (1 mg/kg). Two hundred nanoliters of the viral vector AAV-DJ-hSyn-ChR2-eYFP (5.78 × 10^12^) was bilaterally targeted to the ventrolateral PAG (vlPAG) of postnatal day 21 (P21) to P28 animals [anteroposterior (AP), −3.73; mediolateral (ML), ±0.4; dorsoventral (DV), −2.29; 0° angle] for all electrophysiology studies. For behavioral assays, 200 nl of AAV5.pENN.AAV.CamKII 0.4.Cre.SV40 [7 × 10^12^ viral genomes (vg)/ml] were targeted to the adult vlPAG, as follows: (AP, –4.9; ML, ±0.5; DV, −2.7), and 400 nl of pAAVrg-hSyn-DIO-hM4D(Gi)-mCherry (8 × 10^12^ vg/ml), pAAVrg-hSyn-DIO-hM3D(Gq)-mCherry (7 × 10^12^ vg/ml), or pAAVrg-hSyn-DIO-EGFP (7 × 10^12^ vg/ml) retrogradely expressing virus into the VTA (AP, −3.0; ML, ±0.45; DV, −4.6). Following surgery, the animals recovered for at least 3 weeks to obtain adequate viral expression.

### Hindpaw injections

Mice were anesthetized with 5% isoflurane until a lack of toe-pinch reflex was observed. Then, under 3% isoflurane, the hindpaws of the animals were disinfected with 70% ethanol and a betadine scrub. Following cleaning, either 1% carrageenan in 0.9% saline or saline alone was administered into both hindpaws at 5 μl/g body weight using a 30 ga needle.

### Behavioral testing

Behavioral testing was performed between the hours of 10:00 A.M. to 5:00 P.M. during the lights-on period under either dim or white light conditions in adult animals.

#### von Frey

von Frey testing was performed according to the SUDO (simplified up-down) method (Bonin et al., 2014). Briefly, animals were placed on a mesh screen, and filaments were presented to elicit a withdrawal reflex. An absence of reflex was followed by a smaller filament presentation and vice versa. Animals were exposed to a total of five stimulus presentations per paw.

#### Static hot plate

Animals were placed on a hot plate heated to 55°C, and the latency to hindpaw licking, biting, grooming, or attempted escape from the plate (nocifensive behaviors) was recorded. The trials were terminated at 20 s.

#### Dynamic hot plate

Animals were placed on plate at a warm, but not noxious, temperature (30°C). This temperature was then ramped at a rate of 2.5°/min to 55°C. The trial ended when the animal displayed a nocifensive behavior or when the plate reached 55°C, whichever occurred first.

#### Elevated plus maze

Animals were placed in the center of the elevated plus maze (EPM) and were allowed to roam under low-light conditions for 10 min. The amount of time spent in the open arms were recorded and scored on Biobserve Software. Animals were injected with 1 mg/kg clozapine-*N*-oxide (CNO; Enzo Life Sciences) 15 min before the start of behavioral testing.

#### Light/dark box

Animals were placed in the dark portion of the Light/Dark (LD) box (20 × 40 cm) and allowed to roam for 10 min. The latency to enter the light side of the chamber was recorded. Animals were injected with 1 mg/kg CNO 15 min before the start of the behavioral experiments.

#### Conditioned place preference

Animals were run in dim light conditions. Pretest, animals were placed in the center chamber of a three-chamber apparatus and allowed to explore freely for 15 min. For the next 5 d, animals were conditioned in the morning to 0.9% saline (i.p.) while confined to one side of the chamber and 1 mg/kg CNO (i.p.) while confined to the other side in the afternoons at 30 min per conditioning session. During the post-test, animals freely roamed, drug free, for another 15 min, and time spent on each side of the chamber was measured.

### Brain slice preparation

Horizontal midbrain slices (220 μm) were prepared from 6- to 9-week-old male and female mice 3–6 weeks following viral injection. Animals were deeply anesthetized with drop isoflurane and overdosed with ketamine (37.5 mg/kg) before cardiac perfusion with the following ice-cold oxygenated choline slicing solution (in mm): 110 choline chloride, 25 glucose, 25 NaHCO_3_, 7 MgCl_2_, 11.6 sodium ascorbate, 3.1 sodium pyruvate, 2.5 KCl, 1.25 NaH_2_PO_4_, and 0.5 CaCl2 saturated with 95%O_2_/5% CO_2_, at pH 7.4 and 290–300 mOsm. Brains were rapidly removed and sliced (0.12 mm/s) on a vibratome (model 1200S, Leica). Slices recovered for 10 min at 37°C in the following oxygenated aCSF (in mm): 126 NaCl, 21.4 NaHCO_3_, 2.5 KCl, 1.2 NaH_2_PO_4_, 2.4 CaCl2, 1.0 Mg SO4, 11.1 glucose, and 5 sodium ascorbate. Slices were then held in the same solution at room temperature for up to 8 h until being transferred to a recording chamber.

### Electrophysiology

Brain slices were continuously perfused with oxygenated aCSF (28–32°C) containing 1 μm strychnine and 20 μm bicuculline to block glycine and GABA_A_ receptors, respectively. Dopaminergic neurons were identified via fluorescent labeling in Pitx3-GFP mice (van den Munckhof et al., 2003; Zhao et al., 2004), while GABAergic neurons were identified in slices from vgat-Cre × Ai14 mice (Madisen et al., 2010; Stuber et al., 2015; Root et al., 2018). Whole-cell recordings were performed with patch pipettes (1.8–4 MΩ) containing Kgluc internal solution as follows (in mm): 117 K-gluconate, 2.8 NaCl, 5 MgCl_2_, 2 ATP-Mg, 0.3 GTP-Na, 0.6 EGTA, and 10 HEPES or CsCl internal solution (in mm): 125 CsCl, 2.8 NaCl, 2 MgCl_2_, 2 ATP-Na, 0.3GTP-Na, 0.6 EGTA, and 10 HEPES and voltage clamped at −70 mV. AMPA/NMDA experiment recordings were performed with the CsCl internal solution, and cells were voltage clamped at −70 and +40 mV. In some experiments, QX314 (5 mm) was added to the internal solution. Although GABA_B_ receptors were not blocked, we did not observe synaptic currents at –70 mV, which is consistent with slow GABA_B_R kinetics (Nugent et al., 2009). Additionally, currents were confirmed to be glutamatergic using AP-5 and NBQX where possible, and, as can be seen in Figures 4*A* and 5*A*, there was no residual current in these drugs at +40 mV. Thus, all reported synaptic currents were presumed glutamatergic EPSCs.

#### Stimulation protocols

Channelrhodopin (ChR)-induced optically evoked EPSCs (oEPSCs) were evoked through the microscope objective using full-field light pulses from a white LED (Mightex) controlled by a driver (ThorLabs) and reflected through a 40× water-immersion lens (Polter et al., 2018). All light pulses were of 10 ms duration across experiments to normalize exposure to blue light. Stimulation with pairs of oEPSCs (separated by 50 ms) were separated by 30 s to avoid desensitization of the opsin. The series resistance was monitored continuously during the experiment, and cells were discarded for deviations >15%.

#### AMPA/NMDA ratios

Cells were held at −70 mV, and EPSCs were optically evoked to obtain a stable 10 min baseline. The GluA2-lacking AMPAR-specific antagonist NASPM (20 μm) was then bath applied for 10 min, followed by a 10 min application of the AMPAR antagonist NBQX (10 μm). Cells were then clamped at +40 mV to record the total NMDAR current, followed by a 10 min application of the NMDAR2C/2D antagonist UBP141 (3 μm) and then the NMDAR2B antagonist ifenprodil (10 μm, 10 min). Finally, d-AP-5 (50 μm) was bath applied to confirm that all remaining currents were blocked. Optically evoked EPSCs often had multiple EPSC peaks, and AMPA/NMDA ratios were calculated using the initial peak amplitude from an average of five trials. The responses to NASPM and UBP141 were calculated from an average of five trials at 10 min following bath application of each drug. Drug effects were quantified as the percentage peak EPSC remaining in each drug compared with the EPSC before that drug was applied (10 min prior).

#### Statistics

Results are shown as the mean ± SEM. Significance was determined using one-variable parametric (Student’s *t* tests) or nonparametric comparisons (Mann–Whitney *U* test), depending on data variance. When repeated-measures (RM) two-way ANOVAs were appropriate, they were followed by *post hoc* multiple comparisons where *p* < 0.05. Paired-pulse ratios (PPRs) were calculated using the mean EPSC2 amplitude divided by the mean EPSC1 amplitude for all trials. Coefficient of variation (1/CV^2^) was determined by dividing the EPSC1 mean amplitude squared by the mean variance. AMPA/NMDA ratios were calculated by comparing the basal peak amplitude at −70 mV with its value 10 min following the application of NBQX and dividing this value by the difference between the basal value at +40 mV before d-AP-5. Data were analyzed using GraphPad Prism (9.3.1)

## Results

**Figure 1.**
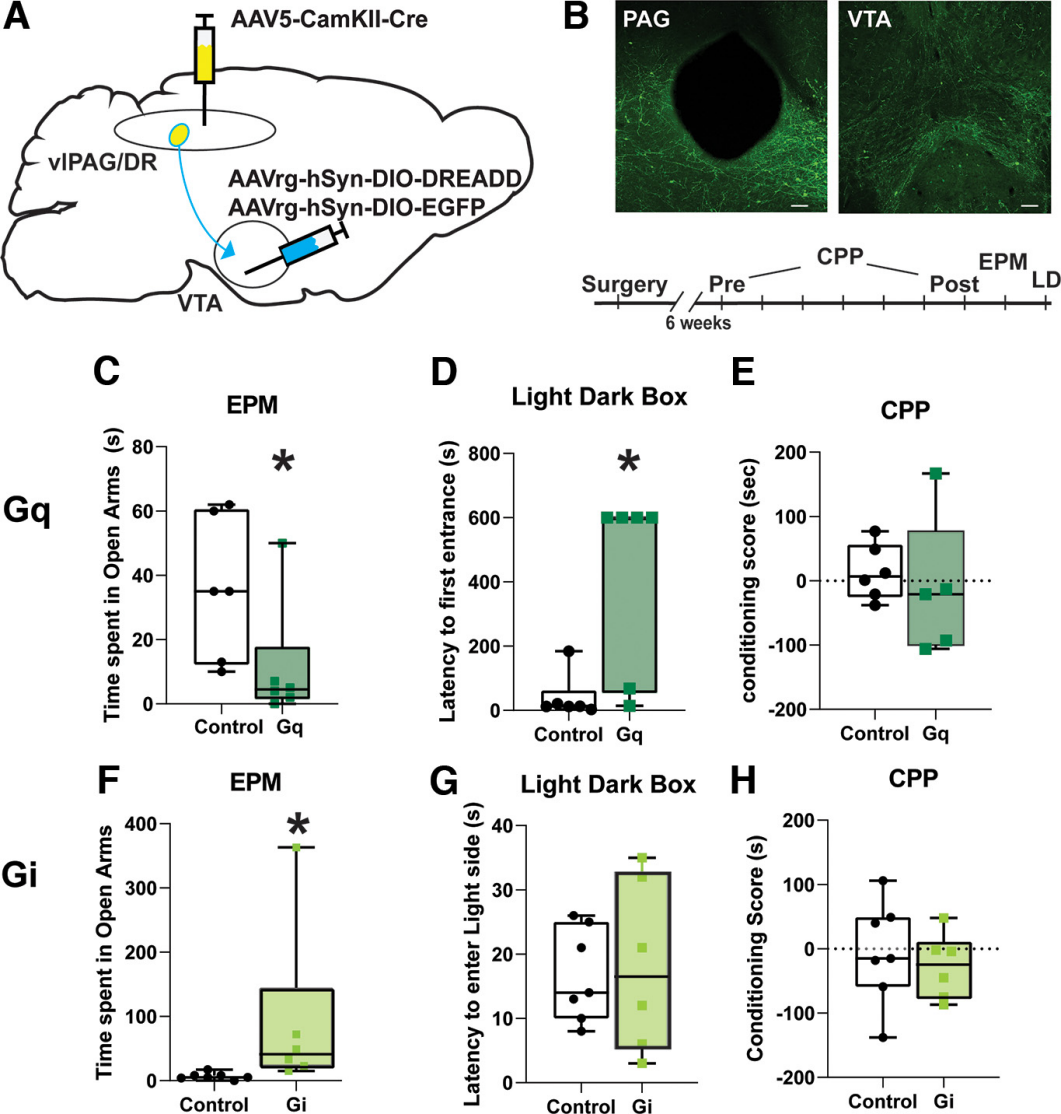
Driving excitatory vlPAG/DR afferents to the VTA reduces exploratory behavior. ***A***, A schematic for the exclusive expression of DREADDs in excitatory PAG/DR projections to the VTA. ***B***, An example of targeting in the PAG (above left) and the expression of terminals in VTA (above right) and the timeline of behavioral assessments (below). In some cohorts, the order of the LD and EPM assays were alternated. ***C***, After CNO administration, mice expressing Gq DREADDS spent less time in the open arms of the elevated plus maze compared with control animals (Mann–Whitney test: *U* = 5, *p* = 0.0216). ***D***, These Gq DREADD-expressing mice also had increased latencies to enter the light side of a light/dark box (*U* = 4, *p* = 0.013). ***E***, CNO administration did not alter CPP scores in these mice (Mann–Whitney test: *U* = 9.5, *p* = 0.355). ***F***, When mice expressing Gi DREADDs in this pathway were exposed to CNO, they significantly increased the time spent in the open arms of the elevated plus maze compared with mice expressing a control virus (Mann–Whitney test: *U* = 2, *p* = 0.0017). ***G***, ***H***, The same mice showed no differences in the time to enter the light side of the LD chamber (Mann–Whitney test: *U* = 20.5, *p* = 0.9755; ***G***) or in the CPP (Mann–Whitney test: *U* = 17, *p* = 0.6282; ***H***). *p < 0.05. Scale bar, 100 μm.

The glutamatergic subpopulation of the vlPAG/dorsal raphe (DR) area has been previously implicated in controlling the behavioral responses to aversive stimuli. Here we first tested the role of driving or inhibiting glutamatergic vlPAG/DR afferents to the VTA in valanced behavior. We used a dual-viral strategy to selectively express either designer receptors exclusively activated by designer drugs (DREADDs) or a reporter in glutamatergic cells of the PAG/DR that project to the VTA (Fig. 1*A*,*B*), and CNO injections (1 mg/kg) were then delivered to exert control over the circuit. pAAV5.pENN.AAV.CamKII 0.4.Cre.SV40 was targeted to the PAG, while pAAVrg-hSyn-DIO-hM4D(Gi)-mCherry, pAAVrg-hSyn-DIO-hM3D(Gq)-mCherry, or pAAVrg-hSyn-DIO-EGFP were directed to the VTA. Driving the vlPAG/DR–VTA circuit with the excitatory Gq-coupled DREADD decreased the amount of time spent in the open arms of EPM (Fig. 1*C*; Mann–Whitney test: *U* = 5 *p* = 0.0216) and increased the latency to enter the light side of an LD box (Fig. 1*D*; Mann–Whitney test: *U* = 4, *p* = 0.013). We also tested for a rewarding/aversive response to driving this pathway using a conditioned place preference (CPP) assay. After a pretest during which mice were allowed to freely explore, mice were conditioned to one side of a three-chamber apparatus with saline and the other side with CNO for 5 d. On the following day, no drug was delivered, and the time spent in each chamber was measured. Place preference scores did not differ between saline and CNO paired chambers (Fig. 1*E*; Mann–Whitney test: *U* = 9.5, *p* = 0.355).

Conversely, DREADD-mediated inhibition of this pathway significantly increased the time spent in the open arms of the EPM (Fig. 1*F*; Mann–Whitney test: *U* = 2, *p* = 0.0017) but did not change the latency to enter the light side of chamber (Fig. 1*G*; Mann–Whitney test: *U* = 20.5, *p* = 0.9755). Similar to the results with the Gq-coupled DREADD, inhibiting this pathway over 2 d also did not affect CPP (Fig. 1*H*; Mann–Whitney test: *U* = 17, *p* = 0.6282). Our data affirm that vlPAG/DR glutamate neurons have a role in an anxiety-like response in mice (Taylor et al., 2019a) and demonstrate that this is mediated through its output to the VTA. Furthermore, our results are consistent with the idea that the vlPAG/DR-to-VTA excitatory afferents might be functionally altered during peripheral injury as pain from inflammatory injury often produces a negative affective state (Hummel et al., 2008; Burek et al., 2022), and we next tested this idea.

### Mice injected with 1% carrageenan have mechanical hypersensitivity 24 h after exposure

**Figure 2.**
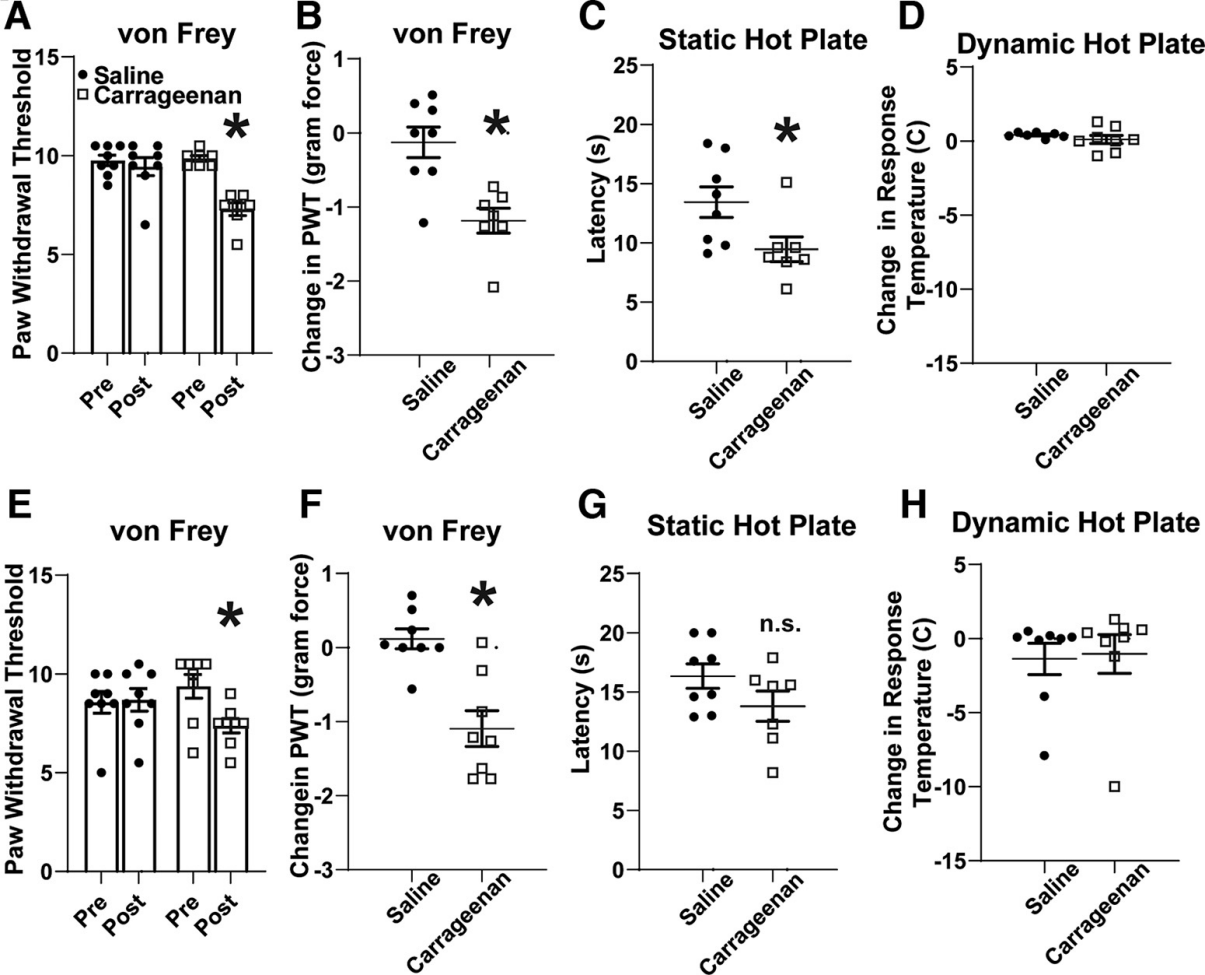
Carrageenan injection into the hindpaws produced mechanical hypersensitivity at 4 and 24 h postinjection. ***A***, There was a significant interaction between hindpaw injection and time as assessed by two-way RM ANOVA (interaction: *F*_(1,13)_ = 17.46, *p* = 0.001). ***B***, Sidak *post hoc* tests revealed that carrageenan-treated mice displayed a paw withdrawal reflex to significantly smaller von Frey filaments compared with saline-treated mice (carrageenan, *p* < 0.001; saline, *p* = 0.655; *N* = 15) with significantly less force required to elicit the paw withdrawal threshold (Student’s *t* test unpaired *t* test, *p* = 0.002). ***C***, ***D***, At 4 h postinjection, carrageenan-treated mice also displayed a significantly reduced latency to nocifensive behaviors on a static hot plate (Student’s *t* test unpaired *t* test, *p* = 0.035; ***C***), but no change in the temperature was required to elicit these behaviors (*p* = 0.342; ***D***). ***E***, ***F***, At 24 h, mice treated with carrageenan had a reduced paw withdrawal [RM two-way ANOVA, *F*_(1,14)_ = 10, *p* = 0.0062, followed by Sidak *post hoc* tests (carrageenan *p* = 0.0256); ***E***] and corresponding to reduced force required to elicit paw withdrawal (Student’s *t* test, *p* = 0.0006; ***F***). ***G***, ***H***, The thermal hypersensitivity was no longer significantly different for static hot plate (Student’s *t* test unpaired *t* test, *p* = 0.14) or for dynamic hot plate (Student’s *t* test unpaired *t* test, *p* = 0.844). *p < 0.05; n.s. > 0.05.

To address functional changes in excitatory PAG–VTA projections following peripheral injury, we leveraged the inflammatory agent λ-carrageenan. When carrageenan is injected into the hindpaw, its maximal nociceptive effect is present at 4 h with resolution at 1–2 d (Benitz and Hall, 1959; Hargreaves et al., 1988; Deuis and Vetter, 2016), and the resultant edema is resolved within a week. We first validated this model in our own hands and assessed thermal and mechanical sensitivity 4 h after injection. In the von Frey assay, carrageenan-treated mice, but not saline-treated mice, had a significantly reduced paw withdrawal threshold (Fig. 2*A*; two-way RM ANOVA: interaction *F*_(1,13)_ = 17.46, *p* = 0.001; and Sidak *post hoc* test: carrageenan, *p* < 0.001; saline, *p* = 0.655; *N* = 15). This directly corresponded to the reduction in mechanical force required for this reflex in carrageenan-treated mice (Fig. 2*B*; unpaired *t* test: *p* = 0.002, *N* = 15). In addition, these animals had thermal sensitivity, with carrageenan-treated animals displaying a reduced latency to nocifensive behaviors on a static hot plate (Fig. 2*C*; unpaired *t* test; *p* = 0.035, *N* = 15). We also used a dynamic hot plate assay but did not observe a change in the temperature required to elicit nocifensive behaviors (Fig. 2*D*; unpaired *t* test; *p* = 0.342, *N* = 15). Together, our C57BL6 mice display mechanical and thermal sensitivity phenotypes 4 h after the injection of a 1% carrageenan solution into the hindpaws.

We also tested the effects of carrageenan on behavior at a 24 h time point. The mechanical hypersensitivity phenotype persisted at 24 h (Fig. 2*E*; two-way RM ANOVA: interaction, *F*_(1,14)_ = 10, *p* = 0.006; and Sidak *post hoc* test: carrageenan, *p* = 0.026; saline, *p* = 0.983; *N* = 16; Fig. 2*F*; unpaired *t* test, *p* < 0.001, *N* = 16), although the thermal sensitivity phenotype resolved at this time point (Fig. 2*G*,*H*; unpaired *t* test; *p* = 0.14a, *p* = 0.844b; *N* = 16), which is consistent with previous reports (Benitz and Hall, 1959; Deuis and Vetter, 2016). Therefore, the mice remain in a state of mechanical hypersensitivity at 24 h.

### Periaqueductal gray afferents to the VTA do not display changes in presynaptic function following carrageenan inflammation

**Figure 3.**
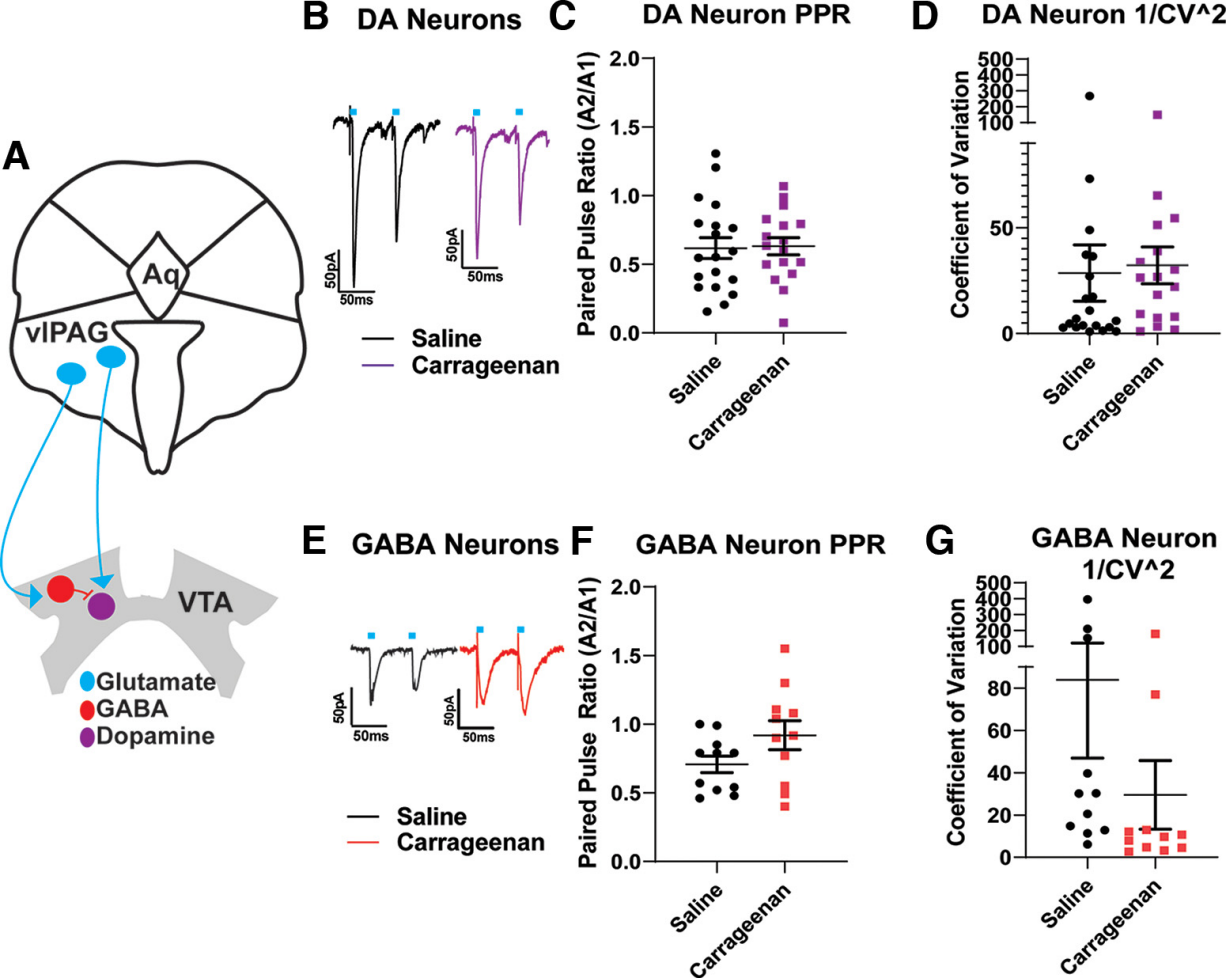
Excitatory periaqueductal gray afferents to the VTA do not display changes in presynaptic function following carrageenan injury. ***A***, A schematic of PAG innervation of the VTA. ***B***, Representative traces of oEPSCs onto VTA DA neurons from saline-treated (black) and carrageenan-treated (magenta) animals. Ten microsecond light pulses are denoted by the blue bar in this and all figures. ***C***, ***D***, When measuring PAG–VTA oEPSCs onto DA neurons, neither PPRs (Student’s *t* test unpaired *t* test, *p* = 0.89) nor the coefficient of variation (1/CV^2^; Student’s *t* test unpaired *t* test, *p* = 0.8266) is changed 24 h following carrageenan injection compared with saline controls. ***E***, Representative traces of oEPSCS from VTA GABA neurons from saline-treated and carrageenan-treated animals. ***F***, ***G***, Cells from carrageenan-treated animals did not exhibit significant changes in paired-pulse ratio (Student’s *t* test unpaired *t* test, *p* = 0.098) or 1/CV^2^ (Student’s *t* test unpaired *t* test, *p* = 0.193) compared with saline controls.

We hypothesized that after inflammatory injury, excitatory synapses on VTA DA neurons may be weakened, and/or excitatory synapses on VTA GABAergic neurons might be strengthened. We first investigated presynaptic function at synapses onto either DA or GABA neurons within the VTA after carrageenan inflammation. To selectively label excitatory PAG afferents, we virally expressed cre-dependent ChR2. In VTA slices, we delivered optical stimulation while recording from fluorescently labeled VTA DA or GABA neurons. When measuring PAG–VTA oEPSCs on DA neurons, neither paired-pulse ratios (PPRs) nor the coefficient of variation were changed at 24 h following inflammatory injury (Fig. 3*C*,*D*; unpaired *t* test: *p* = 0.89c; *p* = 0.8266d; *n* = 36 cells, *N* = 16 animals). Similarly, PAG–VTA oEPSCs on GABA neurons did not show significant changes 24 h following injury, despite a trend toward increased PPR and decreased 1/CV^2^ (Fig. 3*F*,*G*; unpaired *t* test: *p* = 0.098f; *p* = 0.193 g; *N* = 22 cells, *N* = 16 animals). Together, these results suggest that, following acute inflammation, glutamate release probability at synapses on VTA dopamine or GABA neurons is not likely affected.

### Acute inflammation does not alter postsynaptic responses at PAG synapses onto VTA DA neurons

**Figure 4.**
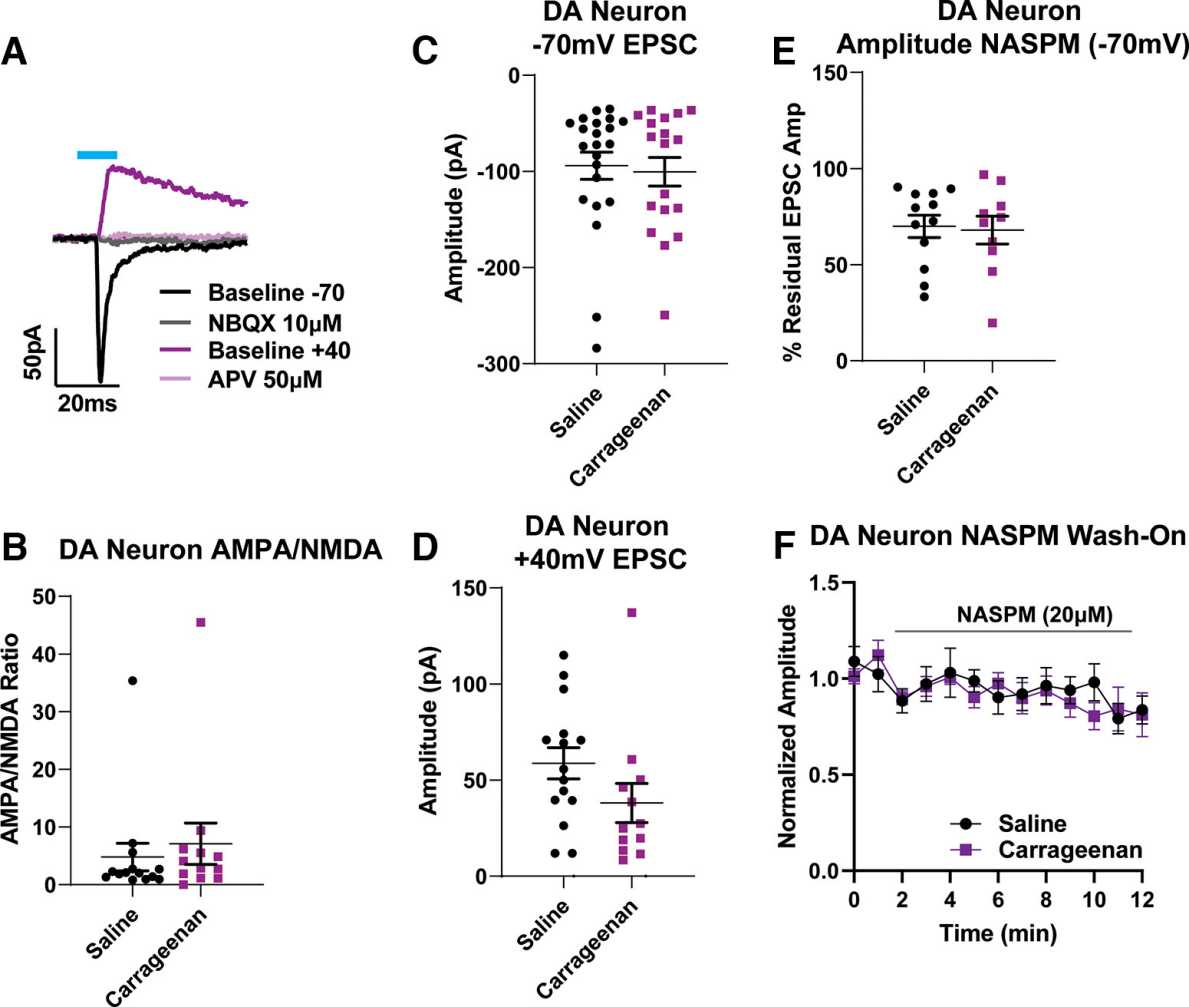
PAG–VTA oEPSCs on DA neurons are unchanged following carrageenan injury. ***A***, Representative example of the optically evoked current protocol used to measure AMPA/NMDA ratios in DA cells. After recording currents at –70 mV (black), NBQX was added (gray); after depolarizing the cell to +40mV (magenta), d-AP-5 was applied (pink; see Materials and Methods). ***B***, AMPA/NMDA ratios at these synapses were not different between saline-treated and carrageenan-treated groups (Student’s *t* test unpaired *t* test, *p* = 0.503). ***C***, Average evoked AMPA currents did not differ between the two groups (Student’s *t* test unpaired *t* test, *p* = 0.76). ***D***, Average evoked NMDAR currents from carrageenan-treated animals were not significantly different compared with saline controls (Student’s *t* test unpaired *t* test, *p* = 0.1213). ***E***, Application of NASPM did not differentially affect oEPSCs at PAG–VTA synapses (Student’s *t* test unpaired *t* test, *p* = 0.83). ***F***, Time course of NASPM on oEPSCs at PAG–VTA synapses.

We next tested whether injury-induced reductions in glutamatergic PAG–VTA synaptic strength on DA neurons may arise instead through changes in postsynaptic glutamate receptors. First, we measured AMPA/NMDA ratios at these synapses, but found no differences between the cells from saline-treated or carrageenan-treated animals (Fig. 4*B*; unpaired *t* test, *p* = 0.503; cells, *N* = 25; animals, *N* = 17). Of note, a subset of these cells had very small NMDA currents despite large AMPA currents, contributing to large AMPA/NMDA ratios. We then investigated the contributions of individual types of glutamate receptors. We found no differences between groups in the amount of current passed at −70 mV (presumed AMPA-mediated currents) or at +40 mV coupled with bath application of NBQX (Fig. 4*C*,*D*: presumed NMDAR currents; unpaired *t* test: *p* = 0.76c; cells, *N* = 40; animals, *N* = 27; p = 0.1213d; cells, *N* = 27; animals, *N* = 25). VTA DA neurons are also known to express calcium-permeable (CP) AMPA receptors (AMPARs; also known as GluA2-lacking AMPARs; Bellone and Lüscher, 2006), which are implicated in forms of synaptic plasticity (Liu and Zukin, 2007). Therefore, we also examined whether there were functional changes in AMPARs at PAG–VTA_DA_ synapses using the CP-AMPAR-specific antagonist NASPM. In the majority of DA cells, EPSCs were reduced by NASPM. However, we observed no differences between cells from saline or carrageenan animals (Fig. 4*E*,*F*; unpaired *t* test, *p* = 0.83; cells, *N* = 22; animals, *N* = 16). Together, these data do not support the hypothesis that acute inflammatory injury alters overall excitatory drive from PAG neurons onto VTA dopamine neurons following an acute inflammatory injury.

### Acute inflammation does not alter postsynaptic responses at PAG synapses onto VTA GABA neurons

**Figure 5.**
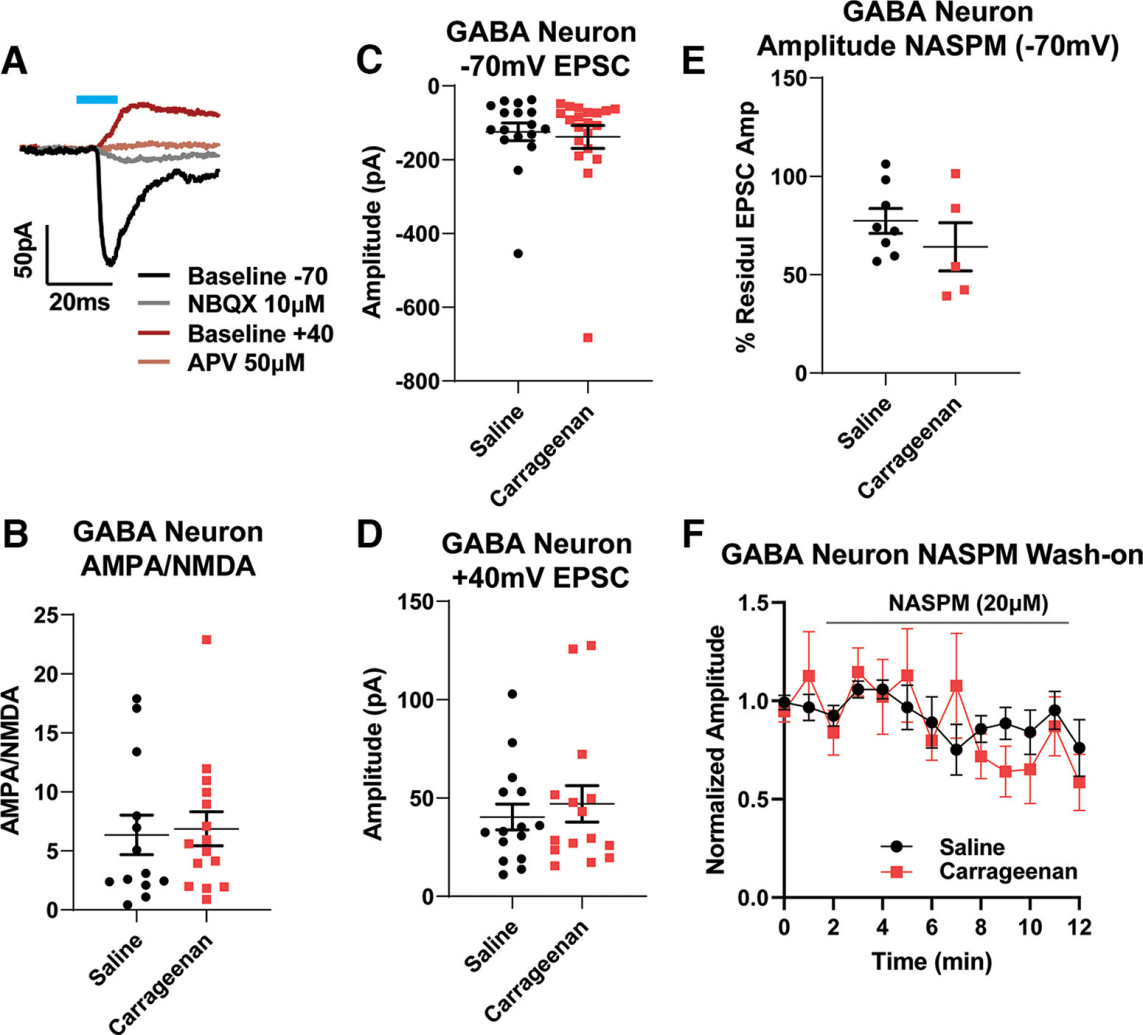
Carrageenan injury does not alter PAG–VTA oEPSCs on GABA neurons. ***A***, Representative example of the optically evoked current protocol used to measure AMPA/NMDA ratios in GABA cells. After recording currents at –70 mV (black), NBQX was added (gray); after depolarizing the cell to +40mV (dark red), APV was applied (brown; see Materials and Methods). ***B***, AMPA/NMDA ratios at these synapses were not different between saline-treated and carrageenan-treated groups (Student’s *t* test unpaired *t* test, *p* = 0.806). ***C***, Average evoked AMPA currents were not different between these two groups (Student’s *t* test unpaired *t* test, *p* = 0.736). ***D***, Average evoked NMDAR currents from carrageenan-treated animals did not differ from saline controls (Student’s *t* test unpaired *t* test, *p* = 0.561). ***E***, Application of NASPM did not differentially affect oEPSCs at PAG–VTA synapses and GABA synapses (Student’s *t* test unpaired *t* test, *p* = 0.31). ***F***, Time course of NASPM on oEPSCs at PAG–VTA synapses.

Many VTA GABA neurons act as local interneurons, effectively controlling the output of DA cells. We therefore next tested whether inflammation results in increased strength of excitatory PAG synapses onto GABAergic VTA neurons. Using the same optogenetic approach to drive excitatory PAG afferents, we examined postsynaptic receptor contributions in identified VTA GABA cells. Again, there were no significant differences between saline-treated and carrageenan-treated cells in AMPA/NMDA ratios, EPSC amplitudes at −70 mV, EPSC amplitudes at +40 mV in the presence of NBQX, or responses to the CP-AMPAR antagonist NASPM (Fig. 5*B–F*; unpaired *t* test: *p* = 0.806b; cells, *N* = 28; animals, N = 15; *p* = 0.736c; cells, *N* = 37; animals, *N* = 23; *p* = 0.561d; cells, *N* = 30; animals, *N* = 15; *p* = 0.31e; cells, *N* = 13; animals, *N* = 7). Together, our data do not support changes in postsynaptic drive at excitatory PAG synapses onto VTA GABA neurons following an acute inflammatory injury.

### Acute inflammation decreases the functional contribution of GluN2D containing NMDARs to PAG–VTA synapses on GABA neurons

**Figure 6.**
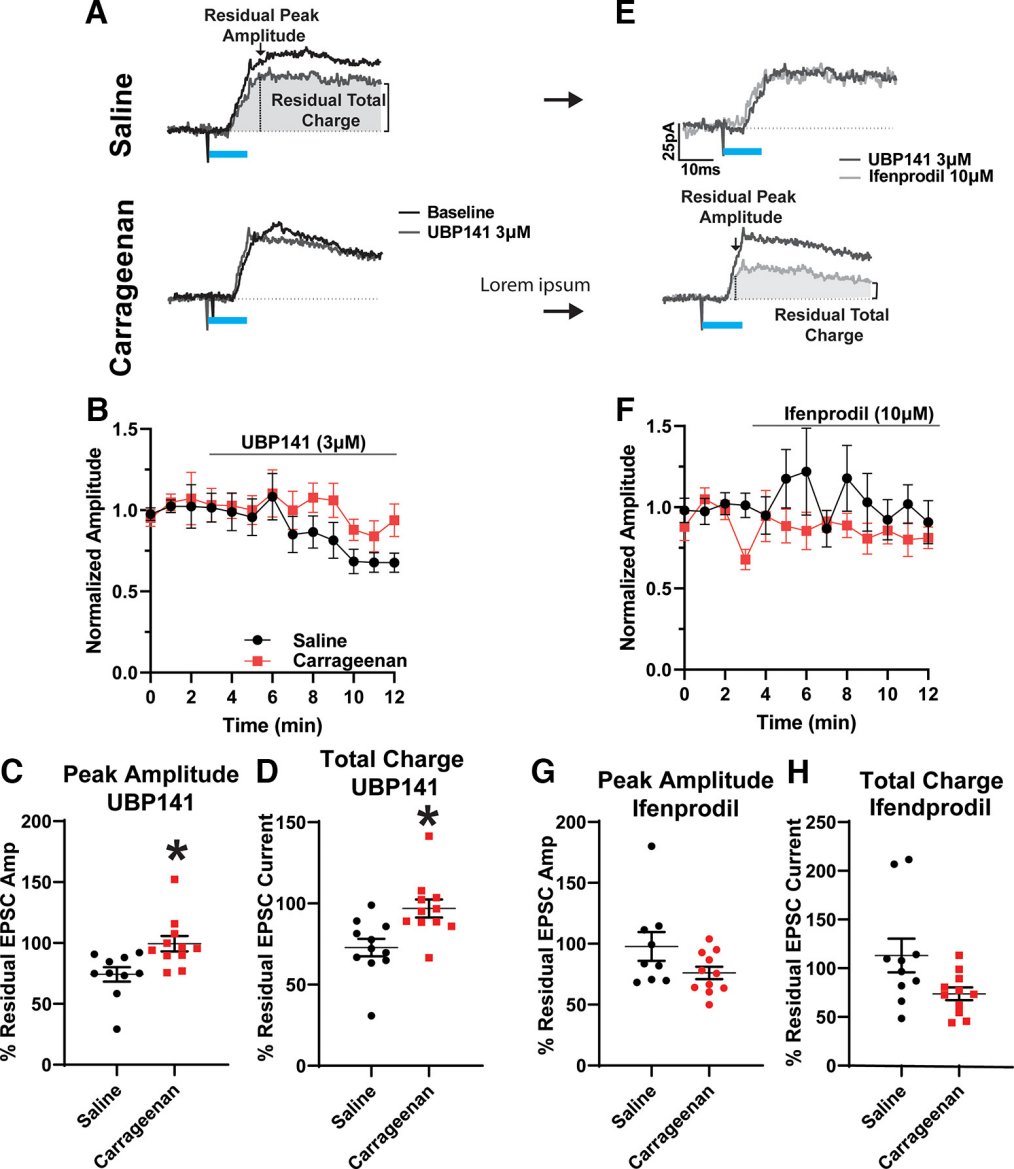
NMDA receptor subunits at PAG synapses on VTA GABA neurons are altered after injury. ***A***, Representative example oEPSCs before and after bath application of UBP141 in GABA neurons from saline-treated (left) or carrageenan-treated (right) animals. ***B***, Time course of isolated peak amplitudes of NMDAR oEPSCs following bath application of UBP141 (3 μm), a GluN2D antagonist, quantified in ***C***. ***C***, Isolated peak NMDAR oEPSCs from saline-treated animals were significantly more responsive to UBP141 (3 μm) compared with cells from carrageenan-treated animals (Student’s *t* test unpaired *t* test, *p* = 0.009). ***D***, The charge transfer of NMDAR oEPSCs of saline-treated animals was significantly more responsive to UBP141 compared with cells from carrageenan-treated animals (Student’s *t* test unpaired *t* test, *p* = 0.0055). ***E***, Example oEPSCs before and after bath application of ifenprodil in the same cells from ***A***. ***F***, Time course of isolated peak NMDAR oEPSCs following bath application of Ifenprodil (10 μm), a GluN2B antagonist, quantified in ***G***. ***G***, There were no significant differences between the peak amplitude oEPSCs from the carrageenan-treated and saline-treated groups in response to ifenprodil (10 μm; Student’s *t* test unpaired *t* test, *p* = 0.121). ***H***, NMDAR oEPSC charge transfer in cells from carrageenan-treated animals was not significantly different in ifenprodil compared with that in cells from saline-treated animals (Student’s *t* test unpaired *t* test: *p* = 0.056). *p < 0.05.

In the previous experiments, we considered NMDARs as classic GluN1/2A or GluN1/GluN2B heteromers. However, midbrain neurons also express the GluN2D subunit into adulthood, evidenced by electrophysiology and *in situ* hybridization (Monyer et al., 1994; Morris et al., 2018). In neurons containing the GluN2D subunits, the Mg^2+^ block is weaker, allowing ionic flux at more negative membrane potentials but passing less Ca^2+^ compared with GluN2B-containing receptors (Hansen et al., 2018). During our experiments recording from VTA GABAergic neurons, we observed measurable inward NMDAR currents at −70 mV, suggesting a contribution of GluN2D subunits at synapses from PAG afferents. Therefore, we next assessed whether the NMDARs at PAG–VTA GABA synapses were differentially sensitive to the GluN2D antagonist UBP141 or GluN2B antagonist ifenprodil. NMDAR currents in GABAergic neurons from carrageenan-treated mice lost their sensitivity to the GluN2D antagonist UBP141 (3 μm) compared with cells from control animals as measured by either peak amplitude (Fig. 6*B*; *p* = 0.0091; cells, *N* = 21; animals, *N* = 15), or total charge (Fig. 6*C*; *p* = 0.0055; cells, *N* = 21; animals, *N* = 15). In contrast, these NMDAR currents were not significantly different in sensitivity to the GluN2B-specific antagonist ifenprodil (10 μm) measured by peak amplitude (Fig. 6*E*; *p* = 0.1210; cells, *N* = 20; animals, *N* = 15) and total charge in response to optogenetic stimulation (Fig. 6*F*; *p* = 0.0556; cells, *N* = 20; animals, *N* = 15), although we noted a trend toward a greater ifenprodil response (EPSC amplitude, *p* = 0.12; EPSC charge, *p* = 0.06). Together, our data suggest that at this time point following acute inflammation, the proportion of NMDARs containing the GluN2B subunit is not significantly changed, but there are fewer NMDARs containing the GluN2D subunit.

## Discussion

### PAG–VTA roles

Through bidirectional modulation of PAG glutamatergic inputs to VTA, we have shown a causal relationship between glutamatergic PAG–VTA and anxiety-like phenotypes both in the EPM and the LD box. Previously, the glutamatergic neurons of the PAG have been highlighted as contributing to anxiety-like phenotypes, but the relevant afferents had not been discovered (Taylor et al., 2019a). We did not, however, observe that chemogenetically activating PAG–VTA excitatory afferents affects place preference (Waung et al., 2019), indicating that stimulation of this pathway is valence neutral. Therefore, driving this afferent population may cause an attenuation of exploratory behavior, which can serve a protective role following inflammatory pain (Burek et al., 2022). Our work represents a step forward in understanding the complex neurocircuit role of the PAG in affective phenotypes.

### Excitatory drive from PAG to VTA DA neurons

In this study, we also present data supporting the idea that excitatory drive from PAG to dopamine neurons is unchanged 24 h after carrageenan inflammatory injury. Presynaptic and postsynaptic metrics associated with excitatory neurotransmission did not reveal any differences between cells from carrageenan-treated and saline-treated mice. Although our results do not support the hypothesis of reduced excitatory neurotransmission following acute inflammation, they are concordant with work suggesting that pain and noxious stimuli generally increases inhibitory neurotransmission onto VTA DA neurons (Markovic et al., 2021; Yang et al., 2021). Our experiments also confirm previous observations showing the presence of GluN2D-containing and Glu2A-lacking glutamate receptors in VTA DA neurons and demonstrating that afferents from the PAG contribute to synapses expressing both receptor subtypes (Jones and Gibb, 2005; Bellone and Lüscher, 2006).

The relevance of circuit-specific complexities cannot be overstated. Of note, in this study we did not patch from the interpeduncular nucleus, rostral linear nucleus, or other rostromedial VTA structures. These structures are thought to contain DA neurons projecting to the medial prefrontal cortex (Beier et al., 2015; Beier et al., 2019), which have increased AMPA/NMDA ratios 24 h following formalin injury (Lammel et al., 2008). Moreover, in our study we have grouped all lateral VTA DA neurons as a single population, despite physiological variation dependent on location within the VTA and projection targets (Lammel et al., 2011; Beier et al., 2015; Morales and Margolis, 2017; Zhang et al., 2017; Huang et al., 2019, 2020). Indeed, in multiple pain models, several groups have shown a reduction in firing rate, AMPA/NMDA ratios, and sEPSCs specifically in the VTA neurons that project to the NAc (Lammel et al., 2011; Ren et al., 2016; Zhang et al., 2017; Markovic et al., 2021). Conversely, VTA neurons projecting to the PFC undergo different alterations such as increased AMPA/NMDA ratios, which is consistent with increased neurotransmission via this pathway following inflammatory pain (Lammel et al., 2008). In light of recently published data concerning other ascending pain pathways to the VTA (Yang et al., 2021), our work highlights that there are parallel streams of ascending pain-relevant information with different magnitudes of injury-induced plasticity.

### Excitatory drive from PAG to VTA GABA neurons

GABAergic neurons in the VTA also may comprise a heterogeneous population, and some of these neurons act locally to inhibit dopamine neurons while others project out of the VTA (Tan et al., 2012; van Zessen et al., 2012; Paul et al., 2018, 2019; Polter et al., 2018; An et al., 2021). Some of our data appear to cluster (Fig. 3*F*,*G*, example), possibly suggesting that PAG excitatory synapses on GABAergic subpopulations may exhibit distinct properties. Further work will be needed to clarify the roles of VTA GABA neuron subgroups in inflammatory injury.

Following acute carrageenan inflammation, we identified a decrease in the currents through GluN2D containing NMDARs at these synapses, without observing other significant alterations in metrics of synaptic function. Within the midbrain, GluN2D (but not Glun2C) subunits are expressed postnatally and into adulthood (Monyer et al., 1994; Wenzel et al., 1996). GluN2D receptors generally exhibit reduced Mg^2+^ block, and do not require depolarization for the influx of calcium, in theory providing a mechanism for tonic NMDAR activity without calcium-dependent inactivation (Gary and Gabriela, 2020). Within midbrain substantia nigra dopamine neurons, NMDARs containing either GluN1/GluN2B or GluN1/GluN2D subunits are expressed postnatally. However, GluN1/Glun2D/GluN2B triheteromers (i.e., NMDAR heterotetramers containing three different types of GluN subunits) develop in these neurons by P21 (Jones and Gibb, 2005; Brothwell et al., 2008; Suárez et al., 2010; Morris et al., 2018). In undifferentiated midbrain neurons, Glun2D subunits can form these triheteromers with either a GluN2A or GluN2B subunit (Dunah et al., 1998), and these conformations are also found in forebrain inhibitory interneurons (von Engelhardt et al., 2015; Perszyk et al., 2016). Our study is the first to examine these in identified synapses onto VTA GABA neurons.

NMDAR-mediated currents at glutamatergic PAG synapses on VTA GABA neurons from carrageenan-treated animals were less sensitive to the GluN2D-biased antagonist UBP141 (3 μm) compared with those from saline-treated mice. This was observed despite unchanged AMPA/NMDA ratios and EPSC amplitudes recorded at either −70 or +40 mV. Morris et al. (2018) suggested that in the absence of triheteromeric GluN2D receptors, midbrain dopamine neurons have increased the expression of GluN1/GluN2B NMDA receptors. In contrast, in VTA GABA neurons we saw a trend toward increased sensitivity to the GluN2B-specific antagonist ifenprodil. Because GluN1/GluN2A/GluN2D triheteromers are found in the midbrain, this perhaps suggests that if VTA GABA neurons undergo subunit substitution following acute inflammation they may incorporate GluN2A, rather than GluN2B, subunits.

This change in NMDAR composition coupled with the consistency of baseline neurotransmission, leads to questions about the functional relevance of GluN2D subunits at these synapses. Changes in Mg^2+^ block and Ca^2+^ entry will directly affect glutamatergic cellular communication. In the hippocampus, Glun2D subunits contribute to synapse maturation (Yamasaki et al., 2014) and the expression of some forms of plasticity as pharmacological antagonism can reduce the magnitude of short-term potentiation or LTP depending on the stimulation protocol (France et al., 2017; Eapen et al., 2021). However, how these manifest in the midbrain are unknown. Clues may come from work on other Ca^2+^-permeable ionotropic glutamate receptors, the GluA2-lacking AMPARs. GluA2-lacking AMPARs and their regulation of Ca^2+^ influx can serve diverse roles, from an excitotoxic signal (Liu and Zukin, 2007) to induction and maintenance of multiple forms of plasticity (Hage et al., 2016), shaping future signal responses including dendritic spine growth and shrinkage.

Midbrain neurons contain glutamatergic synapses on both the spine and shaft of dendrites (Jang et al., 2015; Hage et al., 2016), with NMDARs recruited preferentially to spine heads. There, in conjunction with voltage-gated sodium channels, they can provide a mechanism for changes in synaptically evoked firing patterns in response to dendritic inputs (Hage et al., 2016). Integrating GluN2D NMDAR into this model is expected to allow Ca^2+^ entry across a broader range of membrane potentials, thereby promoting higher-frequency firing at rest. Alternatively, GluN2D-containing NMDARs may serve to increase the frequency of Ca^2+^ transients through these receptors, promoting the maintenance of these synapses; after carrageenan treatment, excitatory synapses from the PAG may be destined for pruning (Liu and Zukin, 2007). These hypotheses remain untested and present an opportunity for future study.

Together, our results show that following injury, the excitatory PAG synapses on GABAergic neurons of the VTA rather than on DA neurons are selectively modulated through the NMDA receptor. As many of these GABA neurons control DA neuron firing via feedforward inhibition, they could contribute to the reported increase in spontaneous GABAergic IPSCs in DA neurons observed during inflammatory pain (Markovic et al., 2021). Further work will be needed to test whether these initial findings at 24 h following injury are exacerbated in a persistent pain model.

## Synthesis

Reviewing Editor: Isabel Perez-Otaño, Instituto de Neurociencias Decisions are customarily a result of the Reviewing Editor and the peer reviewers coming together and discussing their recommendations until a consensus is reached. When revisions are invited, a fact-based synthesis statement explaining their decision and outlining what is needed to prepare a revision will be listed below. The following reviewer(s) agreed to reveal their identity: Manuel Mameli.

Both reviewers found your work investigating how painful states can alter plasticity in the PAG of interest. Particularly, the analysis of NMDA receptor transmission and the connection between plastic and inflammatory events was considered original and a welcome addition to the filed. However, the reviewers had a number of suggestions (particularly defining populations and rates of AAVs infection) that we hope you can address in a revised manuscript.

REVIEWER’S COMMENTS

Reviewer#1:

This is an interesting paper exploring the PAG to VTA pathway, focusing on outputs to both VTA GABA and DA neurons. Broadly, there is not much of an effect on plasticity, with some subtle changes on the inputs to GABA neurons. There are a few areas that when addressed can improve the manuscript.

1) The authors use a camk2-cre approach for the behavioral studies, with a retro-DIO-DREADD and suggest that this is ‘excitatory neurons’. I may have missed it, but is there data supporting this statement? If not, there needs to be some assessment of the cell types that this viral combo expresses in.

2) In the same vein, the authors use AAVDJ-hSyn to express Chr2 and study glutamatergic transmission. They pharmacologically isolate glutamate currents, so the data is sound. They are blocking ionotropic, but not GABAB receptors. I Would not suggest redoing the experiments with GABABB antagonists, but I do think it is useful to address as a limitation.

3) The final summary, suggesting the adaptations in this pathway relate to pain-induced affect, does not really appear to be supported by data, I would suggest removing this and focusing more on what the data is telling.

4) very minor question: For the study of glutamate transmission... why are the authors using a high chloride intracellular?

Reviewer#2:

In this work the authors assess the impact of noxious experience onto synapses from the periaqueductal grey to neurons of the midbrain including dopamine and GABA cells. Using optogenetic assisted stimulation and electrophysiology in acute brain slices, the authors discover that synaptic plasticity takes place after acute carrageenan inflammation at excitatory PAG-to-GABA neurons synapses but not onto dopamine neurons. This plasticity expresses via loss of the NR2D subunit of the NMDA receptor.

This piece of work is interesting and I find the use of the different transgenic mice and viral approaches good to identify correctly the cell populations of interest. However, certain aspects need to be strengthened. See points below.

Major points:

1- The authors use a combination of viral vectors to express DREADD (Excitatory and inhibitory) at PAG to VTA. The combination of which viral construct is unclear. Which AAV is used for retrograde expression of Dreadds? This is not indicated and in addition the labeling of the midbrain should be shown. What rate of infection in the PAG do the authors observe? A quantification of the rate of recombination should be provided. This basic information is required in order to assess the solidity of the behavioural data.

In Panel D 2 mice seem to have very low latency compared to the rest of the cohort. Is this a technical failure, or part of the biological efficacy of DREADDs?

2- The recording sample traces show large variability. The authors indeed for panel B and E have chosen to rather different traces not really matching the average. Did the authors consider to correct the territorial distribution in their recordings in the VTA ? It is known that medial and lateral VTA are projecting to different structures, and this variability may hide some important biological factor.

In the coefficient of variation analysis few cells completely result off the scale. This may actually be normal in a large population, but in this case this is driven by only few cells. Did the author consider to check their recordings, noise analysis and other parameters?

3- For experiments in figure 4 and 5 it would be informative to see a timecourse of the effect of NASPM on synaptic current. This is also valid for the use of UBP141 product and ifenprodil.

Is the current voltage relationship of I-NMDA different between experimental groups?

4- One point that remain unknown is the potential repercussions of this plasticity. Do the authors think this change in subunit composition may change activity properties of long-term plasticity? Although not mandatory, I think few experiments aiming to solve these questions may strengthen the message of this work.

## Author Response

Response to Reviewers

We thank the reviewers for their careful reading of the manuscript and their constructive suggestions. We hope to have addressed their concerns and believe the manuscript is better as a result

REVIEWER’S COMMENTS

Reviewer#1:

This is an interesting paper exploring the PAG to VTA pathway, focusing on outputs to both VTA GABA and DA neurons. Broadly, there is not much of an effect on plasticity, with some subtle changes on the inputs to GABA neurons. There are a few areas that when addressed can improve the manuscript.

We appreciate the reviewer’s constructive comments.

1) The authors use a camk2-cre approach for the behavioral studies, with a retro-DIO-DREADD and suggest that this is ‘excitatory neurons’. I may have missed it, but is there data supporting this statement? If not, there needs to be some assessment of the cell types that this viral combo expresses in.

This is based upon a literature showing that CaMK2 does not overlap with GAD indicating that these are excitatory neurons (Jones 1994, Benson 1992), and this same strategy has also been used in the PAG as well (Zhu 2019). These PAG neurons have also been functionally confirmed as glutamatergic (Barcomb 2022).

2) In the same vein, the authors use AAVDJ-hSyn to express Chr2 and study glutamatergic transmission. They pharmacologically isolate glutamate currents, so the data is sound. They are blocking ionotropic, but not GABAB receptors. I Would not suggest redoing the experiments with GABABB antagonists, but I do think it is useful to address as a limitation.

Thank you for addressing this. We have now included mention of this: (lines 184-187). “Although GABAB receptors were not blocked, we did not observe synaptic currents at -70mV consistent with slow GABABR kinetics (Nugent et al., 2009). Additionally, currents were confirmed to be glutamatergic using AP-5 and NBQX where possible, and as can be seen e.g. in Figures 4A and 5A there was no residual current in these drugs at +40mV. Thus, all reported synaptic currents were presumed glutamatergic EPSCs.”

3) The final summary, suggesting the adaptations in this pathway relate to pain-induced affect, does not really appear to be supported by data, I would suggest removing this and focusing more on what the data is telling.

Thank you for this suggestion. We have edited the final summary to refocus on our electrophysiological findings.

4) very minor question: For the study of glutamate transmission... why are the authors using a high chloride intracellular?

There is no special justification for the high Cl- in this set of experiments, although it avoids junction potentials between the electrode and AgCl ground. At the bench, this solution facilitated working on multiple projects using a single pipette solution.

Reviewer#2:

In this work the authors assess the impact of noxious experience onto synapses from the periaqueductal grey to neurons of the midbrain including dopamine and GABA cells. Using optogenetic assisted stimulation and electrophysiology in acute brain slices, the authors discover that synaptic plasticity takes place after acute carrageenan inflammation at excitatory PAG-to-GABA neurons synapses but not onto dopamine neurons. This plasticity expresses via loss of the NR2D subunit of the NMDA receptor.

This piece of work is interesting and I find the use of the different transgenic mice and viral approaches good to identify correctly the cell populations of interest. However, certain aspects need to be strengthened. See points below.

We appreciate the reviewer’s helpful comments.

Major points:

1- The authors use a combination of viral vectors to express DREADD (Excitatory and inhibitory) at PAG to VTA. The combination of which viral construct is unclear. Which AAV is used for retrograde expression of Dreadds? This is not indicated and in addition the labeling of the midbrain should be shown. What rate of infection in the PAG do the authors observe? A quantification of the rate of recombination should be provided. This basic information is required in order to assess the solidity of the behavioural data.

We apologize for the inadvertent lack of clarity on this key point. We targeted an AAV5 serotype to locally express cre within the PAG (AAV5.pENN.AAV.CamKII 0.4.Cre.SV40). We then used Addgene’s pAAVrg serotype targeted to the VTA to retrogradely express the cre-dependent constructs (pAAVrg-hSyn-DIO-hM4D(Gi)-mCherry, pAAVrg-hSyn-DIO-hM3D(Gq)-mCherry, or pAAVrg-hSyn-DIO-EGFP). We have now included an image of midbrain labelling to illustrate our approach (shown below), and altered the text and figures to clarify the use and titer of the viruses. While we did not specifically calculate infection rate or recombination rate, rAAV can express one week following injection (Shen 2013) and persist up to 6 months in brainstem (McCown 1999) and thus our time point of six weeks provides a point at which the virus should be present within all our cells of interest, and we would not expect differences in infection rate between the treatment groups due to their identical capsids and similar gene insert sizes. Similar combinatorial approaches have been used frequently in recent years to pinpoint a specific afferent input (Yizhar 2011, Fenno 2011, Fenno 2014, Gore 2013, Eagle 2020).

In Panel D 2 mice seem to have very low latency compared to the rest of the cohort. Is this a technical failure, or part of the biological efficacy of DREADDs?

We thank the reviewer for their question. We excluded mice that were mistargeted from the behavioral analyses and all mice were given the same dose of DREADD ligand, so we do not believe this is directly related to our independent variable (i.e. DREADD efficacy), but may instead reflect the inherent variability present in behavioral assessments.

2- The recording sample traces show large variability. The authors indeed for panel B and E have chosen to rather different traces not really matching the average. Did the authors consider to correct the territorial distribution in their recordings in the VTA ? It is known that medial and lateral VTA are projecting to different structures, and this variability may hide some important biological factor.

Thank you for this comment. Our traces were chosen to be of similar sizes to one another, but they did not reflect the mean for each example experiment. We have now replaced them with traces approximating the mean responses.

We do appreciate the complexities of the VTA’s structure, as mentioned in the discussion. In horizontal slices, the delineations between VTA subnuclei are often difficult to distinguish. The PBP is the largest subnucleus with the best visibility, so although we did not specifically account for it most of these cells are from the lateral VTA.

In the coefficient of variation analysis few cells completely result off the scale. This may actually be normal in a large population, but in this case this is driven by only few cells. Did the author consider to check their recordings, noise analysis and other parameters?

We thank the reviewer for this point. We are aware of these clusters and prior to submission we directly examined each piece of data for such quality checks. These large values arise from occasional failures on the first stimulus pulse from those cells, and can be an issue in analysis of this measure. Even so, removal of these points did not affect the result.

3- For experiments in figure 4 and 5 it would be informative to see a timecourse of the effect of NASPM on synaptic current. This is also valid for the use of UBP141 product and ifenprodil.

We thank the reviewer for these suggestions and have added four panels of these time courses in Figures 4, 5, and 6.

Is the current voltage relationship of I-NMDA different between experimental groups?

In our UBP experiments we see a relatively small change of ∼20% in NMDAR current. While significant, it is a modest effect size when passing maximal current (∼<30pA at +40mV) at these sampled synapses. Perhaps not surprisingly then, when looking at voltage ranges when the Mg2+ blockage is still in play we could not detect significant differences in the NMDAR I-V curves between treatment groups. It remains likely that the amount of Ca2+ entering the neurons may still be significantly different even at these more physiological potentials, although it is masked in the total NMDAR current values.

4- One point that remain unknown is the potential repercussions of this plasticity. Do the authors think this change in subunit composition may change activity properties of long-term plasticity? Although not mandatory, I think few experiments aiming to solve these questions may strengthen the message of this work.

We appreciate this comment. This question is fascinating but beyond the scope of this current study. Some work in rat hippocampus from the Collingridge lab indicates that NMDAR subunit plasticity may contribute to facets of both STP and LTP as measured by electrically evoked field recordings. In their hands, antagonism of Glun2D can reduce the magnitude of LTP and to a lesser extent the magnitude of STP following a burst stimulation protocol (Eapen 2021).

The discussion has been altered to include mention of this point (lines 442-446). “In the hippocampus Glun2D subunits contribute to synapse maturation (Yamasaki et al., 2014) and the expression of some forms of plasticity as pharmacological antagonism can reduce the magnitude of STP or LTP depending on the stimulation protocol. (Eapen et al., 2021 and France et al., 2017), However, how these manifest in the midbrain are unknown.”

Barcomb, K., Olah, S.S., Kennedy, M.J. and Ford, C.P. Properties and modulation of excitatory inputs to the locus coeruleus. J Physiol. (2022), https://doi-org.stanford.idm.oclc.org/10.1113/JP283605

Benson DL, Isackson PJ, Gall CM, Jones EG. Contrasting patterns in the localization of glutamic acid decarboxylase and Ca2+/calmodulin protein kinase gene expression in the rat central nervous system. Neuroscience. 1992;46(4):825-49. doi: 10.1016/0306-4522(92)90188-8. PMID: 1311814.

Eagle AL, Manning CE, Williams ES, Bastle RM, Gajewski PA, Garrison A, Wirtz AJ, Akguen S, Brandel-Ankrapp K, Endege W, Boyce FM, Ohnishi YN, Mazei-Robison M, Maze I, Neve RL, Robison AJ. Circuit-specific hippocampal ΔFosB underlies resilience to stress-induced social avoidance. Nat Commun. 2020 Sep 8;11(1):4484. doi: 10.1038/s41467-020-17825-x. PMID: 32901027; PMCID: PMC7479591.

Eapen AV, Fernández-Fernández D, Georgiou J, Bortolotto ZA, Lightman S, Jane DE, Volianskis A, Collingridge GL. Multiple roles of GluN2D-containing NMDA receptors in short-term potentiation and long-term potentiation in mouse hippocampal slices. Neuropharmacology. 2021 Dec 15;201:108833. doi: 10.1016/j.neuropharm.2021.108833. Epub 2021 Oct 9. PMID: 34637787; PMCID: PMC8607330.

Fenno L, Yizhar O, Deisseroth K. The development and application of optogenetics. Annu Rev Neurosci. 2011;34:389-412. doi: 10.1146/annurev-neuro-061010-113817. PMID: 21692661; PMCID: PMC6699620.

Fenno LE, Mattis J, Ramakrishnan C, Hyun M, Lee SY, He M, Tucciarone J, Selimbeyoglu A, Berndt A, Grosenick L, Zalocusky KA, Bernstein H, Swanson H, Perry C, Diester I, Boyce FM, Bass CE, Neve R, Huang ZJ, Deisseroth K. Targeting cells with single vectors using multiple-feature Boolean logic. Nat Methods. 2014 Jul;11(7):763-72. doi: 10.1038/nmeth.2996. Epub 2014 Jun 8. PMID: 24908100; PMCID: PMC4085277.

Gore BB, Soden ME, Zweifel LS. Manipulating gene expression in projection-specific neuronal populations using combinatorial viral approaches. Curr Protoc Neurosci. 2013;65(435):4.35.1-20. doi: 10.1002/0471142301.ns0435s65. PMID: 25429312; PMCID: PMC4242517.

Jones EG, Huntley GW, Benson DL. Alpha calcium/calmodulin-dependent protein kinase II selectively expressed in a subpopulation of excitatory neurons in monkey sensory-motor cortex: comparison with GAD-67 expression. J Neurosci. 1994 Feb;14(2):611-29. doi: 10.1523/JNEUROSCI.14-02-00611.1994. PMID: 8301355; PMCID: PMC6576801.

Nugent FS, Niehaus JL, Kauer JA. PKG and PKA signaling in LTP at GABAergic synapses. Neuropsychopharmacol 2009 34:1829-42.

McCown TJ, Xiao X, Li J, Breese GR, Samulski RJ. Differential and persistent expression patterns of CNS gene transfer by an adeno-associated virus (AAV) vector. Brain Res. 1996 Mar 25;713(1-2):99-107. doi: 10.1016/0006-8993(95)01488-8. PMID: 8724980.

Shen S, Horowitz ED, Troupes AN, Brown SM, Pulicherla N, Samulski RJ, Agbandje-McKenna M, Asokan A. Engraftment of a galactose receptor footprint onto adeno-associated viral capsids improves transduction efficiency. J Biol Chem. 2013 Oct 4;288(40):28814-23. doi: 10.1074/jbc.M113.482380. Epub 2013 Aug 12. PMID: 23940044; PMCID: PMC3789977.

Yizhar O, Fenno LE, Davidson TJ, Mogri M, Deisseroth K. Optogenetics in neural systems. Neuron. 2011 Jul 14;71(1):9-34. doi: 10.1016/j.neuron.2011.06.004. PMID: 21745635.

Zhu H, Xiang HC, Li HP, Lin LX, Hu XF, Zhang H, Meng WY, Liu L, Chen C, Shu Y, Zhang RY, Zhang P, Si JQ, Li M. Inhibition of GABAergic Neurons and Excitation of Glutamatergic Neurons in the Ventrolateral Periaqueductal Gray Participate in Electroacupuncture Analgesia Mediated by Cannabinoid Receptor. Front Neurosci. 2019 May 17;13:484. doi: 10.3389/fnins.2019.00484. PMID: 31156369; PMCID: PMC6533898.
